# Increased transmission and incidence of syphilis in southern Sweden 2007–2022

**DOI:** 10.1017/S095026882510085X

**Published:** 2025-12-23

**Authors:** Niclas Winqvist, Edith Almqvist, Julia Andreasson, Gunnel Henriksson, Per Hagstam, Annika Johnsson, Anton Reepalu

**Affiliations:** 1 Clinical Infection Medicine, Department of Translational Medicine, Lund University, Sweden; 2 Department for Infectious Diseases, Skåne University Hospital, Sweden; 3 Department of Clinical Microbiology, Infection Prevention and Control, Skåne, Sweden; 4 Division of Medical Microbiology, Department of Laboratory Medicine, Lund Uni, Sweden; 5 Regional Office of Communicable Disease Control and Prevention, Region Skåne, Sweden; 6 Centre for Sexual Health, Skåne University Hospital, Sweden

**Keywords:** Syphilis, Sweden, Epidemiology, Risk groups, sexually transmitted infections (STI), men who have sex with men (MSM)

## Abstract

Syphilis has re-emerged as a public health threat during the 21st century, and updated knowledge of the epidemic and its drivers is needed to halt this worrying development. We present data on the incidence of syphilis in the south Swedish region Skåne from 2007 to 2022 to determine the burden of disease, changes in risk groups, as well as routes for testing. To get a picture of the burden of syphilis, both early (notifiable) syphilis and cases of non-notifiable (late) symptomatic syphilis were included in this register-based study. Mann–Kendall trend analysis (MK) was used to determine statistical significance over time. In all, 584 cases of syphilis were included in the study. The overall syphilis incidence in Skåne increased from 3.1 cases/100000 population in 2007 to 6.3 in 2022 (MK z-stat: 2.57; p = 0.010). The highest increase in absolute numbers was among men who have sex with men (MSM), from eight cases annually in 2007 to 62 in 2022, but also for heterosexually transmitted men and women, with under ten cases yearly from 2007 through 2019 to 22 cases in 2022. We also found that transmission within Sweden was common, indicating that local measures are needed to curb this epidemic.

## Bullet points

- The overall syphilis incidence in Skåne increased from 3.1 cases/100000 population in 2007 to 6.3 in 2022.
- The increase was mainly driven by men who have sex with men, but heterosexual men and women were also affected from 2020 onwards.
- Syphilis transmission commonly occurred within Sweden, indicating that measures to curb this epidemic need to be taken locally.

## Introduction

Syphilis has re-emerged as a public health threat in many high-income countries around the world in the first decades of the 21st century [1]. Several countries report incidence data reaching levels not seen since the 1950s [1–3]. As a consequence of increased incidence, a worrying observation is also that the number of children born with congenital syphilis has risen steeply in some countries in the European Union and in the USA, exemplified by 3,700 reported cases in the USA in 2022, a 1,000% increase compared with 2012 [4, 5].

Syphilis, caused by the spirochaete *Treponema pallidum* subspecies *pallidum*, is a mainly sexually transmitted disease, but it can also be transmitted by blood products and vertically from mother to child during pregnancy [6]. Sexual transmission is considered possible during the initial year following infection, whereafter sexual transmission is rare. Syphilis diagnosed during the initial year from infection is classified as early disease, with further categorization into primary, secondary, and early latent syphilis depending on symptoms and/or clinical findings. These early stages of syphilis are considered the main drivers of syphilis epidemics primarily through sexual transmission. Additionally, the risk of mother-to-child transmission is highest during these early stages of syphilis. Late syphilis (more than one year from the time of infection) is primarily categorized as either late latent or tertiary syphilis, but untreated secondary syphilis can be symptomatic even at this stage. Importantly, ocular, oto-, and neurosyphilis can occur at any time following infection, both early and late [7].

In Sweden, syphilis incidence reached an all-time low during the late 1990s but began to rise during the first years of the new millennium [8]. In 2012, Velicko and Unemo presented the most recent in-depth analysis of Swedish national syphilis data, indicating that the syphilis epidemic in Sweden from 2007 to 2011 seemed to have stabilized with the major burden being among men who have sex with men (MSM) [9]. Since then, national surveillance data from the Public Health Agency of Sweden indicate a renewed increase in notifiable (early) syphilis, with an increased incidence from 2.1 cases per 100,000 persons in 2012 to 5.1 cases per 100,000 in 2022 [10]. In the same report, MSM remains the group with the highest burden of early syphilis, with 77% of cases reported from this group.

According to the Swedish Communicable Disease Act, syphilis is considered dangerous to public health, which entails a mandatory contact tracing and notification to the Regional Office for Communicable Disease Control and Prevention as well as to the Public Health Agency of Sweden [11]. Furthermore, medical examination, diagnostic procedures, and treatment for persons with suspected or confirmed syphilis are free of charge. The indications for a clinician to order a diagnostic test for syphilis can be categorized into symptom-driven investigations or asymptomatic screening. The asymptomatic screening can in turn be provider-initiated or patient-initiated. Patient-initiated asymptomatic testing is dependent on awareness of syphilis as a sexually transmitted infection (STI) so that a history of possible syphilis risk exposure triggers the person to ask for a syphilis test. Provider-initiated testing also depends on a sufficient level of awareness of the current syphilis epidemiology to appropriately suspect syphilis and order a diagnostic test. In most countries, there are guidelines for whom to screen for syphilis, regardless of symptoms, targeting either high-risk groups such as MSM or situations where a missed syphilis diagnosis could have detrimental downstream effects such as for pregnant women and blood donors. The current guidelines from the Swedish Society for Dermatology and Venereal Diseases recommend screening for syphilis in those with a clinical or epidemiological suspicion of syphilis; MSM; sexual contacts abroad or via transactional sex; people living with HIV; and unprotected sex with multiple partners [12]. Tracing and testing of sexual contacts to a person diagnosed with early syphilis is also recommended. In 2016, screening for syphilis was discontinued in asylum seekers in most regions of Sweden due to low yield. Screening during pregnancy (gestation week 4–12) and before blood donation is still recommended. A recent adjustment to the syphilis screening recommendations has been implemented since the roll-out of pre-exposure prophylaxis for HIV (PrEP), which was launched in Sweden 1 November 2018. For those on PrEP, syphilis screening is mandated every three months [13].

Given the recent rise in reported syphilis cases, the aim of this study was to analyse the current epidemiology and burden of early and symptomatic syphilis in Skåne region, southern Sweden, from 2007 through 2022. Incidence dynamics and possible changes in risk group composition were analysed, and the number of cases diagnosed by the different indications for syphilis testing was evaluated.

## Methods

This retrospective study was performed in Skåne region, the southernmost region of Sweden. The population of Skåne region was 1.2 million in 2007 and 1.4 million in 2022 (approximately 13% of the Swedish population, 90% urban population). Malmö is the largest municipality in Skåne with a population of 350,000 inhabitants in 2022, about 25% of Skåne’s population. Malmö is also connected to Copenhagen in Denmark by a bridge since 2000. For this study, all cases of either early (notifiable) or symptomatic syphilis (regardless of duration) diagnosed and treated in Skåne region from 1 January 2007 until 31 December 2022 were eligible for inclusion. The Swedish (and most international) definition of notifiable disease stage changed in 2017 from two years from the estimated time of infection to one year from the estimated time of infection. The corresponding definitions before and after 2017 were used in this study. The International Statistical Classification of Diseases and Related Health Problems (ICD-10) diagnosis codes A50–53, A65, H57–58, I98.0, K67.2, M03.1, M73.1, N29.0, N74.2, and O98.1 were used to identify individuals with symptomatic syphilis eligible for inclusion.

All cases of notifiable syphilis in Skåne are reported to the Regional Office for Communicable Disease Control and Prevention (Smittskydd Skåne) in accordance with the Swedish Communicable Disease Act. Data from Smittskydd Skåne were obtained along with an extract from the regional medical record system (Melior), where data from all patients with any of the above-mentioned ICD-10 diagnosis codes are kept. An individual medical chart review in Melior and linked microbiological database was thereafter performed with cross-checking of the data from Smittskydd Skåne and data collection for those without notifiable yet symptomatic syphilis. During the chart review, the following exclusion criteria were applied: inaccurate ICD-10 coding (defined as the absence of positive syphilis serology and polymerase chain reaction test, and no description of syphilis in the medical record); treatment for syphilis outside Skåne region; and lack of access to medical records as a result of blocked medical records either due to privacy concerns or due to temporary identification numbers in the notification report. Those excluded because of blocked records due to privacy concerns were deemed more likely to represent actual cases of syphilis and were therefore included in the overall incidence estimates on age group and assigned sex. The rationale behind this assumption was that most blocked records belonged to patients seeking care at sexual health clinics in contrast to those with inaccurate ICD-10 coding.

The variables extracted from the notification reports and medical records were: year of diagnosis; stage of syphilis disease; presumptive route of transmission and country of infection; indication for syphilis testing; age at diagnosis; assigned sex at birth; gender identity; sexual orientation; HIV infection; self-reported previous history of syphilis; previous history of venereal chlamydia and/or gonorrhoea (self-reported or documented in the microbiological database); current diagnosis of venereal chlamydia and/or gonorrhoea at the time of syphilis diagnosis (documented in the microbiological database); current PrEP use; and year of starting PrEP.

In cases where discrepancies were found between the notification report and the medical record, the data from the medical record were kept. Presumptive geographical region of infection was categorized using UN development groups [14] to make the presentation of data comprehensible and to ensure individual integrity. For the statistical analysis, only one stage of syphilis was considered per case. If signs of more than one stage were present, the case was classified as ocular, oto-, and neurosyphilis, over secondary syphilis, over primary syphilis. The variable ‘indication for syphilis testing’ was categorized into the following indications: symptomatic, contact tracing, provider-initiated, patient-initiated, and unknown.

The medical chart review was performed by two investigators (EA and JA). To assess possible observer bias during chart review, cross-checking of fifty randomly selected cases was performed, with no discrepancies found. Late latent syphilis was not considered in this study. At times, it was not possible to determine the duration of a syphilis infection with certainty. In this study, we classified cases with unknown duration as early if the treating clinician had made a notification report. Hence, some cases of late latent syphilis could have been included in this study.

Descriptive analysis was undertaken for all variables, including the number of cases, medians, and ranges for each category of all variables. Population-based incidence was calculated with regard to age group and assigned sex per year. To explore statistical significance, the Mann–Kendall trend test (MK) was used to detect monotonic trends over time [15]. Statistical significance was set at p < 0.05.

To explore variations in patterns of testing, de-identified data on syphilis testing were collected from Skåne Region Clinical Microbiological Laboratory database. Besides results (positive/negative/inconclusive), data on age and sex at diagnosis and health provider were assembled.

Ethical approval for this study was obtained on 25 September 2023 from the Swedish Ethical Review Authority (dnr 2023–05141-01) with a waiver of the requirement for informed consent due to the retrospective design of the study.

## Results

**Figure 1.**
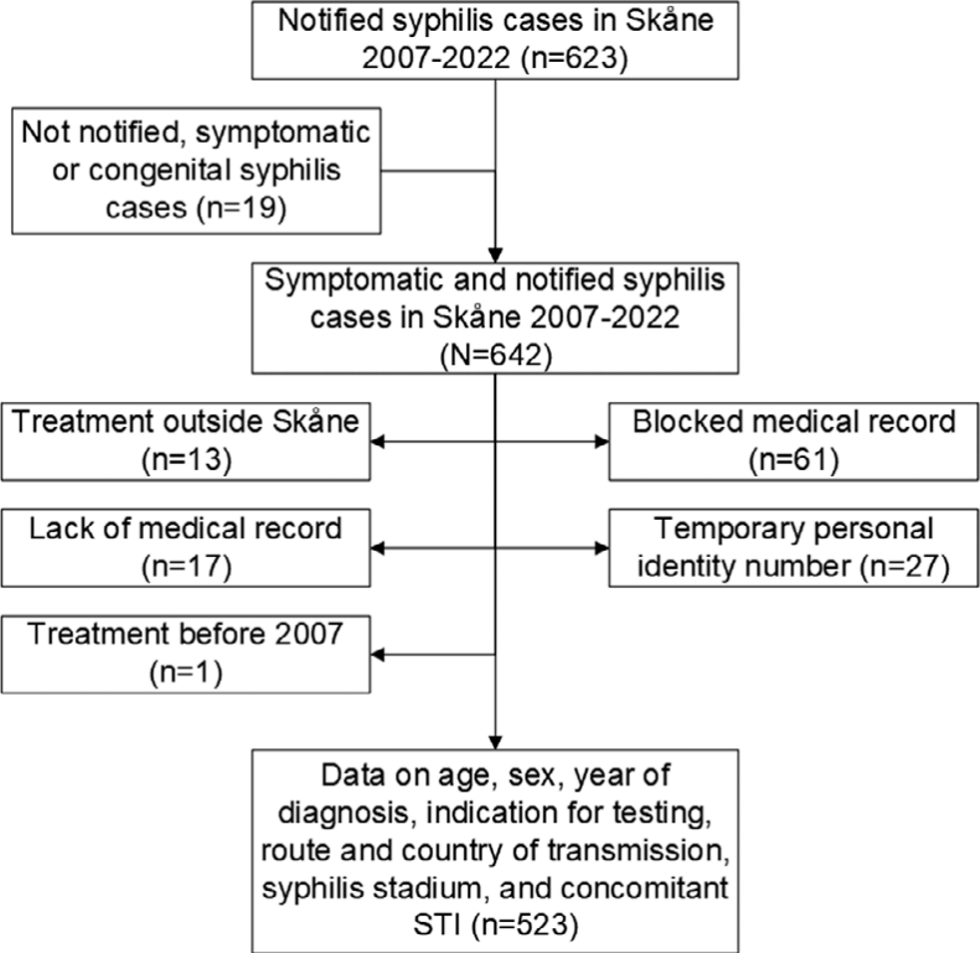
Flowchart of included and excluded syphilis cases in Skåne 2007–2022. STI, sexually transmitted infection.

A flowchart of eligible and excluded study patients is presented in Figure 1. In all, full information was retrieved for 523 out of 642 unique syphilis cases. Of the 523 included cases with early syphilis, 74 cases were among individuals with more than one (range 2–6) episode of syphilis during the study period, resulting in 449 individual patients contributing data. For an additional 59 patients with blocked medical records, representing 61 cases, some basic information such as sex and age was retrieved and added in trend calculations on these specific variables. No significant differences were recorded between cases with and without blocked journals regarding sex and age distribution. Case characteristics are presented in Table 1.

**Table 1.** Descriptive data on all included syphilis cases in Skåne region from 2007 to 2022

	All cases	Cases without blocked journals
**Number of cases**	584	523
**Sex (%)**		
Men	499 (85.4)	450 (86.0)
Women	85 (14.6)	73 (14.0)
**Gender identity (%)**		
Cisgender	–	516 (98.7)
Transgender	–	7 (1.3)
**Median age at diagnosis, years (IQR)**	37 (29–48)	38 (29–49)
**Age (%)**		
0–14 years	3 (0.5)	3 (0.6)
15–19 years	18 (3.1)	16 (3.1)
20–24 years	56 (9.6)	53 (10.1)
25–34 years	167 (28.6)	148 (28.3)
35–44 years	157 (26.9)	131 (25.0)
45+ years	183 (31.3)	172 (32.9)
**Contributed cases per individual (%)**		
1 case	508 (87.0)	449 (85.8)
2 cases	55 (9.4)	53 (10.1)
3 cases	13 (2.2)	13 (2.5)
4 cases	4 (0.7)	4 (0.8)
5 cases	3 (0.5)	3 (0.6)
6 cases	1 (0.2)	1 (0.2)
**Stage (%)**		
Primary	–	182 (34.8)
Secondary	–	115 (22.0)
Latent	–	173 (33.1)
Neuro, ocular, otic	–	21 (4.0)
Congenital	–	4 (0.8)
Unknown primary, secondary, or latent	–	28 (5.3)
**Place of transmission (%)**		
Sweden	–	283 (54.1)
Europe/North America	–	174 (33.3)
Africa and Western, Central and Southern Asia	–	15 (2.9)
Eastern/Southeastern Asia	–	22 (4.2)
Latin America, Caribbean, and Oceania	–	11 (2.1)
Unknown	–	18 (3.4)
**Route of transmission (%)**		
Sexually same sex	–	383 (73.2)
Sexually other sex	–	99 (18.9)
Other	–	10 (1.9)
Unknown	–	31 (5.9)
**Test indication (%)**		
Symptomatic	–	256 (48.9)
Contact tracing	–	144 (27.5)
Provider-initiated	–	70 (13.4)
Patient-initiated	–	43 (8.2)
Unknown	–	10 (1.9)

IQR, interquartile range.

**Figure 2.**
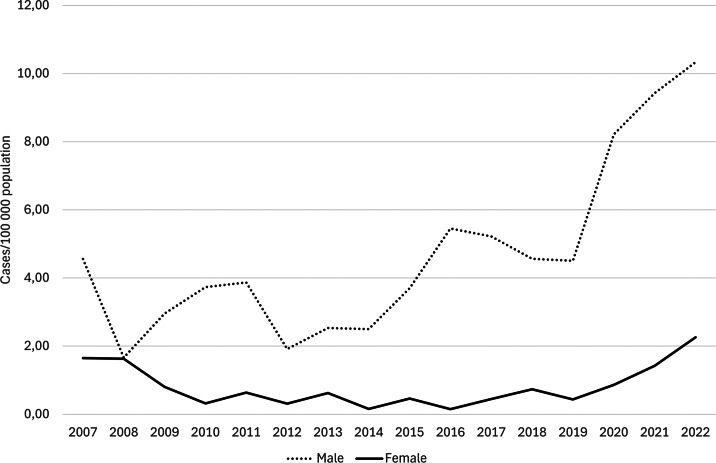
Incidence of syphilis by sex in Skåne region 2007–2022.

The overall syphilis incidence in Skåne increased from 3.1 cases/100000 population in 2007 to 6.3 in 2022 (MK z-stat: 2.57; p = 0.010). For men, it increased from 4.6 to 10.3 cases per 100,000 population in 2007–2022, an increase of 127% (MK z-stat: 2.93; p < 0.01). From 2007 through 2016, the number of cases among women decreased from ten to one case per year, but the number then increased to ten in 2021 and 16 in 2022, giving an increase in incidence to 2.26 cases per 100,000 population in 2022. Although no statistically uniform increasing trend for the entire study period 2007–2022 was seen (p = 1.00), Figure 2 clearly shows an increasing trend from 2019 onwards.

The epidemic dynamics by age group are depicted in Supplementary Figure 1. Minor changes occurred from 2007 through 2015 whereafter an increase was seen among individuals aged 35 to 44 years in 2016. In 2017, there was an upsurge in the age groups 20–34 years, and from 2020 onwards, an increase in all age groups above 19 years was obvious, most prominent among those 20–24 years.

The increase among men was to a great extent attributable to MSM, where the number of cases per year increased by a factor of eight from 7 to 56 during the study period (p < 0.001), but also cases with reported heterosexual transmission increased from four in 2007 to 22 in 2022 (p = 0.017). As the denominator was unknown, the incidence by sexual practice could not be calculated.

**Figure 3.**
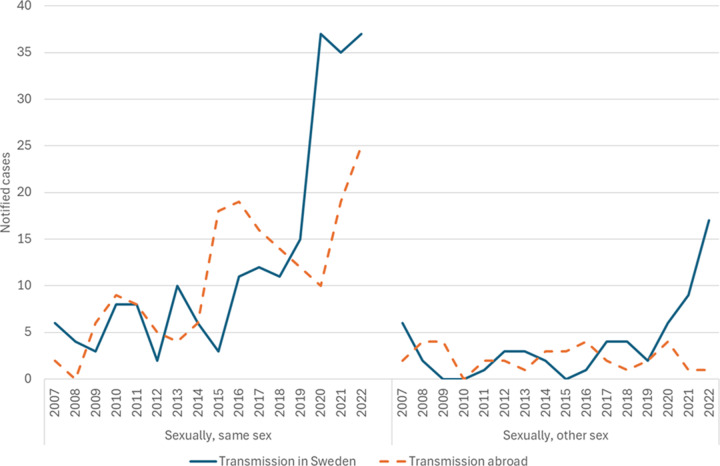
Number of notified syphilis cases per reported country of infection and mode of transmission in Skåne region, 2007–2022.

Out of 523 syphilis cases in Skåne region in 2007–2022, 505 (96.6%) had stated the country of probable transmission. Out of these, 283 (56.0%) were infected in Sweden (Table 1). Over the study period, about half of the cases were transmitted in Sweden on a yearly basis, with an exception for 2015–2016 when more cases (2015: 88.5%; 2016: 66.7%) were infected abroad. From 2020 onwards, the fraction of local transmissions increased to 65.9%. divided by mode of transmission, there was an increase in the number of notified cases with local transmission both among MSM and declared heterosexual contact (Figure 3).

The distribution of notified cases by syphilis stage is presented in Supplementary Figure 2. The increasing trends of primary, secondary, and early latent stages were all statistically significant using MK trend test (z: 4.01, p < 0.001; z: 3.55, p < 0.001; z: 3.12, p < 0.01, respectively), whereas the increasing trend of the potentially disabling ocular, oto-, and neurosyphilis did not reach statistical significance (z: 1.66, p = 0.097). However, with 13 cases during the last four years of the study period compared to eight during the first 12 years, the increase in incidence was at a factor of 4.5 (0.05 cases/100000 person-years vs. 0.23/100000 person-years). Evenly distributed over the study period, four cases of congenital syphilis were reported. Of these, one child was born in Sweden from a mother of foreign origin, whereas the remaining three, aged one, eight, and eight years, respectively, were not born in Sweden and were diagnosed and treated during the study period for sequelae of previously untreated congenital syphilis.

During the first years of the study period, 2007–2016, the number of annually tested individuals in Skåne region increased from 27,401 to 41,848, mostly due to health examination among asylum seekers. In 2016, most regions in Sweden, including Skåne region, abandoned syphilis screening in asylum seekers due to low efficacy. After that, the number of individuals tested yearly returned to the levels seen before 2014, with even lower numbers during the COVID-19 pandemic. Women were to a much greater extent tested for syphilis compared to men, but this surplus was entirely attributable to the screening of pregnant women.

Whereas provider- and patient-initiated testing did not result in an increase in the number of cases over the study period, contact tracing and symptomatic testing yielded significantly more positive cases over time (Supplementary Figure 3).

The trends for syphilis cases with regard to HIV status are shown in Supplementary Figure 4. Both for HIV-negative and HIV-positive patients, the trends were statistically significant, but the increase was more prominent among HIV-negative subjects, especially after 2016 (z: 3.83, p < 0.001 and z: 2.73, p < 0.01 respectively).

At their first syphilis diagnosis, 247 out of 449 individuals (55.0%) had a history of previous STI (Supplementary Table 1). Most of them (92.3%) had had either gonorrhoea or *Chlamydia trachomatis* infection or both, and 15.1% had a confirmed HIV infection. Two additional cases of HIV occurred after the first syphilis diagnosis, both before PrEP was introduced. Sixty-nine patients (15.4%) had a concomitant STI together with a diagnosis of their first syphilis infection and 13 patients out of 74 (17.6%) with repeated syphilis infections had concomitant STI.

**Figure 4.**
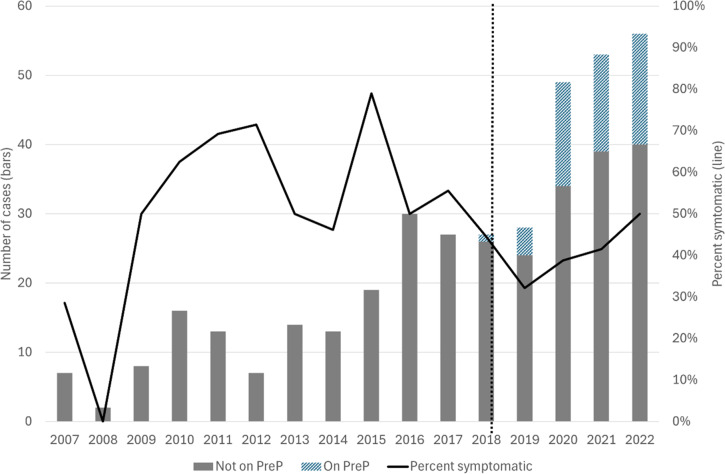
Number of notified syphilis cases among men who have sex with men divided by provided pre-exposure prophylaxis for HIV (PreP; bars) and per cent symptomatic cases (solid line) per year, 2007–2022. PreP was introduced in November 2018 (dotted line).

Out of 252 cases of syphilis recorded since the introduction of PrEP in Skåne region in November 2018, 49 (19.4%) were on PrEP at the time of diagnosis. Using MSM and previous STI as a proxy for eligibility for PrEP, another 40 (15.9%) cases were notified in this group, whereas 163 (64.7%) cases were recorded in patients not eligible for PrEP (Supplementary Table 1). Figure 4 shows the number of syphilis cases among MSM divided by PrEP prescription and year. In the same graph, the proportion of symptomatic cases shows an increase after the introduction of PrEP in Skåne region. Out of 49 MSM on PrEP between 2019 and 2022, 19 (38.8%) were reported as having symptoms. Among 86 HIV-negative MSM without PrEP during the same period, 40 (46.5%) were symptomatic (odds ratio: 1.37; 95% confidence interval 0.67–2.81; *P* = 0.38).

## Discussion

In this registry-based, longitudinal study of Skåne region from 2007 through 2022, we found an increasing incidence of early and symptomatic syphilis. Syphilis remained most common among men, particularly MSM, but a trend of increased syphilis among women could also be seen from 2019 onwards. This is of particular concern given that the increased incidence among women was most pronounced for those aged 25–34 years, which in turn corresponds with the mean age of pregnancy in Sweden [16]. In our study, congenital syphilis remained rare over the entire study period. However, recent reports from both the USA and Japan have shown dramatic increases in rates of congenital syphilis [17, 18]. This has led to the implementation of additional syphilis screening at later stages during pregnancy in areas with particularly high incidence among women of childbearing age [19]. Although current rates of syphilis among women in Skåne may not warrant further intensification of syphilis screening, it is important to note that population-based incidence data may miss subgroups at higher risk. Continued surveillance and identification of possible subgroups at particularly high risk of acquiring syphilis during pregnancy is necessary to maintain the incidence of congenital syphilis as low as possible.

In regard to both demographic constitution and the national distribution of reported cases of STI, Skåne region would be representative of Sweden [20]. Our finding of a statistically significant increase in syphilis incidence over time among men is also in line with previously reported national surveillance data from Sweden and elsewhere. The increased incidence was primarily seen among MSM with an eightfold increase in annually reported cases during the study period. Yet, heterosexual transmission increased by a factor of four during the same period, indicating that the spread of syphilis among men was not limited to the group of MSM. This is in contrast with Swedish national surveillance data, where the incidence of syphilis among heterosexual men has remained low [21]. On a European Union level, a similar increase among heterosexual men has been noted during 2022–2023 [5]. In England, the syphilis notification rate among heterosexual men and women in 2023 showed a continuation of the annual increase seen prior to the COVID-19 pandemic, with a temporary decline from 2020 to 2022 explained by lower testing [22]. Why our data from Skåne seem to follow another pattern compared to national Swedish data is not known, but the increase highlights a lack of awareness of the importance of testing for syphilis among the heterosexual population.

Coinciding with the travel restrictions imposed due to the COVID-19 pandemic in 2020 [23], the proportion of syphilis cases with reported local transmission started to increase substantially. A similar transmission trend was seen for both heterosexual and homosexual modes of transmission. Although travel restrictions in most parts of the world were lifted in 2021–2022, no signs of a decreased proportion of cases where the infection was acquired locally in Sweden could be seen in this study. The same increase in total notification pattern from 2018 through 2022 was observed in 20 out of 28 reporting countries within the European Union [24]. Mathematically, smaller sexual networks impose higher risk of getting infected once syphilis is introduced, but this cannot be concluded from our study setup. Whether or not this increase was attributable to restrictions following the COVID-19 pandemic, this clearly shows that local preventive measures are needed to curb the syphilis epidemic, while keeping in mind that syphilis infections acquired abroad remain relatively common among MSM.

To capture the full burden of syphilis disease during the study period, we included all cases of early (notifiable) syphilis as well as cases of late, symptomatic (non-notifiable) syphilis. Whereas we found that primary, secondary, and early latent syphilis significantly increased over time, congenital syphilis remained rare. Ocular, oto-, and neurosyphilis are of particular concern when it comes to estimating the burden of syphilis disease. Lack of timely diagnosis and effective treatment can lead to irreversible sequelae with substantial impact on the quality of life of the affected individual. Although we did not find a significantly increased incidence of these entities over time, the number of cases per year peaked in 2021, with six reported cases. The incidence was comparable to that reported in a recent Danish study reporting 0.3 cases of neurosyphilis per 100,000 population 2015–2021 [25]. Presently, many clinicians in primary care and other medical specialties most likely to first encounter patients with these potentially debilitating conditions are not aware that neurological manifestation of syphilis, including ocular and otic manifestations, can occur at any stage of syphilis and are not the same as tertiary syphilis [7]. Indeed, it was noted during data collection that several patients sought healthcare multiple times before receiving a syphilis diagnosis. As incidence of syphilis now is rising, educational efforts among medical care providers are urgent to improve the knowledge of the return of the great imitator.

To establish a syphilis diagnosis, the first step is to order the test, either by clinical suspicion or screening an at-risk person. Contact-tracing has played a fundamental role in keeping STIs under control [26]. In our study, we report a large number of cases diagnosed through contact-tracing, showing that this path to a syphilis diagnosis remains highly relevant. Further, we found a large and increasing proportion of cases tested due to symptomatic disease. This trend was exaggerated following the introduction of PrEP. For people prescribed PrEP in Sweden, a quarterly syphilis testing is mandated [13]. In theory, this should increase the number of cases found through provider-initiated, asymptomatic testing [27]. However, in addition to the mandated syphilis screening, people prescribed PrEP are also encouraged to seek care and get tested in case of suspected symptomatic syphilis in between the mandated screening appointments. Symptomatic syphilis is most contagious and therefore prioritized for prompt diagnosis and treatment, yet campaigns aimed at improved early symptom recognition have not been shown to be effective [28]. We found that both provider-initiated and patient-initiated asymptomatic screening yielded relatively few cases with only a minor increase in the latter part of the study period.

It is also obvious that the increased incidence of syphilis means that many people now present with syphilis as their first STI. This was exemplified in our study by the fact that less than 15% of syphilis cases had concomitant chlamydia and/or gonorrhoea and two out of five patients (40.0%) had not had chlamydia and/or gonorrhoea before getting syphilis. Increasing the general awareness of syphilis among care providers and the general public seems urgent.

A particular strength of the current study was the region-wide design aimed to encompass all cases of both early syphilis and symptomatic syphilis regardless of duration in Skåne region 2007–2022. Furthermore, a detailed medical chart review allowed for more in-depth data collection as well as cross-checking of data routinely collected through the mandated notification reports. However, this strategy also conferred a limitation since cases that could not be verified by medical chart review were excluded. To minimize the impact on the overall incidence estimates, we decided to include those not accessible due to blocked medical records. Another limitation was the unknown country of birth, which prevented important insights into the populations affected and which could have been targeted for prevention measures.

In conclusion, we report an increasing incidence of syphilis in Skåne region in 2007–2022 mainly driven by MSM yet also affecting heterosexual men and women, principally from 2020 onwards. Importantly, local transmission dominates among heterosexuals, with both local and international transmission being common among homosexuals. Further, we show that a high number of cases were found through contact tracing but also from investigations of symptomatic disease. These conditions underline the importance of strengthening local measures such as testing availability and contact tracing procedures. Increased knowledge about syphilis both among health care providers and among sexually active people is needed to ensure adequate preventive measures as well as prompt diagnosis and treatment.

## Supporting information

10.1017/S095026882510085X.sm001 Winqvist et al. supplementary material Winqvist et al. supplementary material

## Data Availability

The participants of this study did not give written consent for their data to be shared publicly, so due to the sensitive nature of the research, supporting data are not available.

## Supplementary material

The supplementary material for this article can be found at http://doi.org/10.1017/S095026882510085X.
