# Supplementary material for: Increased transmission and incidence of syphilis in southern Sweden 2007–2022
Source: Epidemiol Infect. 2025 Dec 23;154:e1. doi: 10.1017/S095026882510085X (PMC12766532; doi:10.1017/S095026882510085X)
Supplement: Winqvist et al. supplementary material [file S095026882510085Xsup001.pdf]

Increased transmission and incidence of syphilis in Southern Sweden 2007-2022

Winqvist N et al.

Supplement

	Cases	Individuals
**Number of subjects 2007-2022**	523	449
**Previous history of STI* (%)**		
None	202 (38.6)	202 (45.0)
Syphilis	67 (12.8)	46 (10.2)
Chlamydia trachomatis	172 (32.9)	118 (26.3)
Gonorrhoea	165 (31.5)	110 (24.5)
HIV	113 (21.6)	68 (15.1)
Unknown	4 (0.8)	4 (0.9)
**Concomitant STI with syphilis diagnosis* (%)**		
None	441 (84.3)	380 (84.6)
Chlamydia trachomatis	40 (7.6)	32 (7.1)
Gonorrhoea	39 (7.5)	31 (6.9)
HIV	14 (2.7)	12 (2.7)
Unknown	4 (0.8)	4 (0.9)
**Number of subjects 2019-2022**	**252**	**214**
**PreP (% among cases 2019-2022)**		
On PreP at diagnosis	49 (19.4)	42 (19.6)
Eligible but not on PreP**	40 (15.9)	35 (16.4)
Not eligible for PreP	163 (64.7)	137 (64.0)

STI: Sexually transmitted infections, PreP: Pre-exposure prophylaxis

*) More than one STI possible

**) MSM with previous STI and diagnosis after 2018

Supplemental table 1. Previous and concomitant STI and PrEP use among people diagnosed with syphilis.

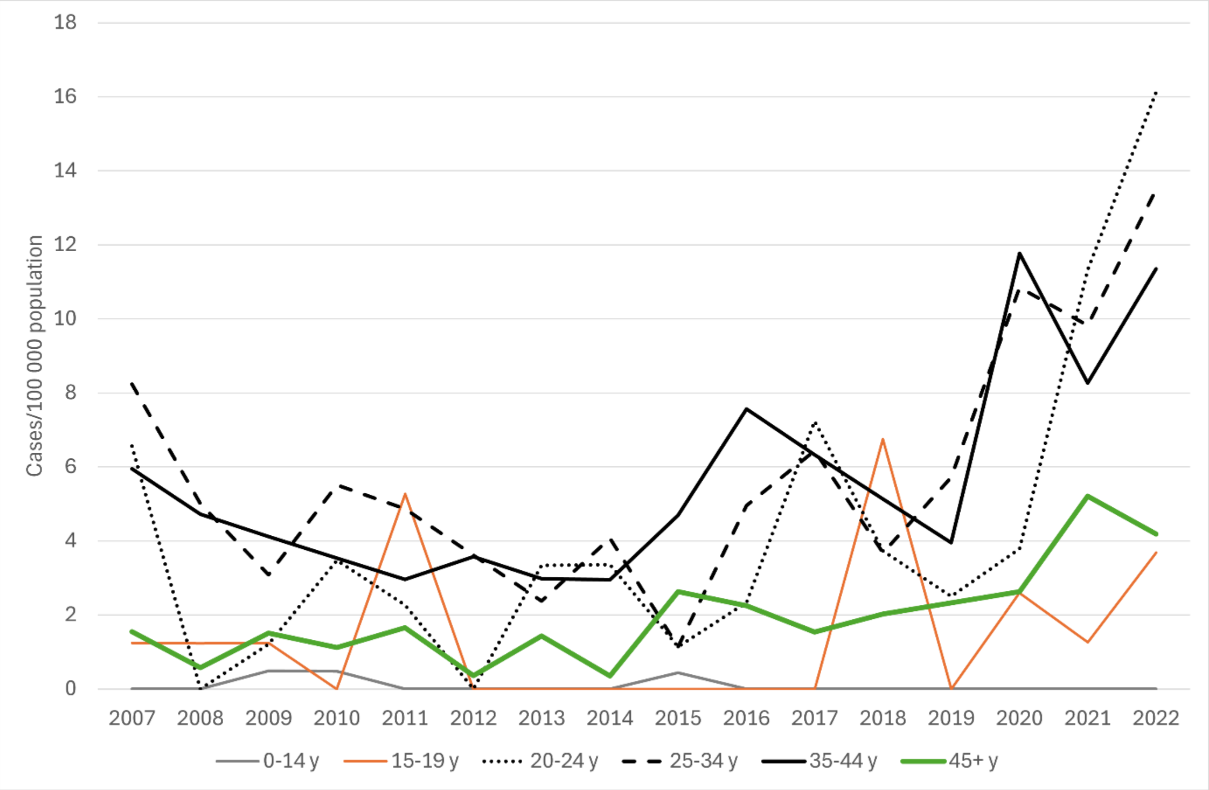

Supplemental figure 1. Syphilis incidence per age group in Skåne region 2007-2022

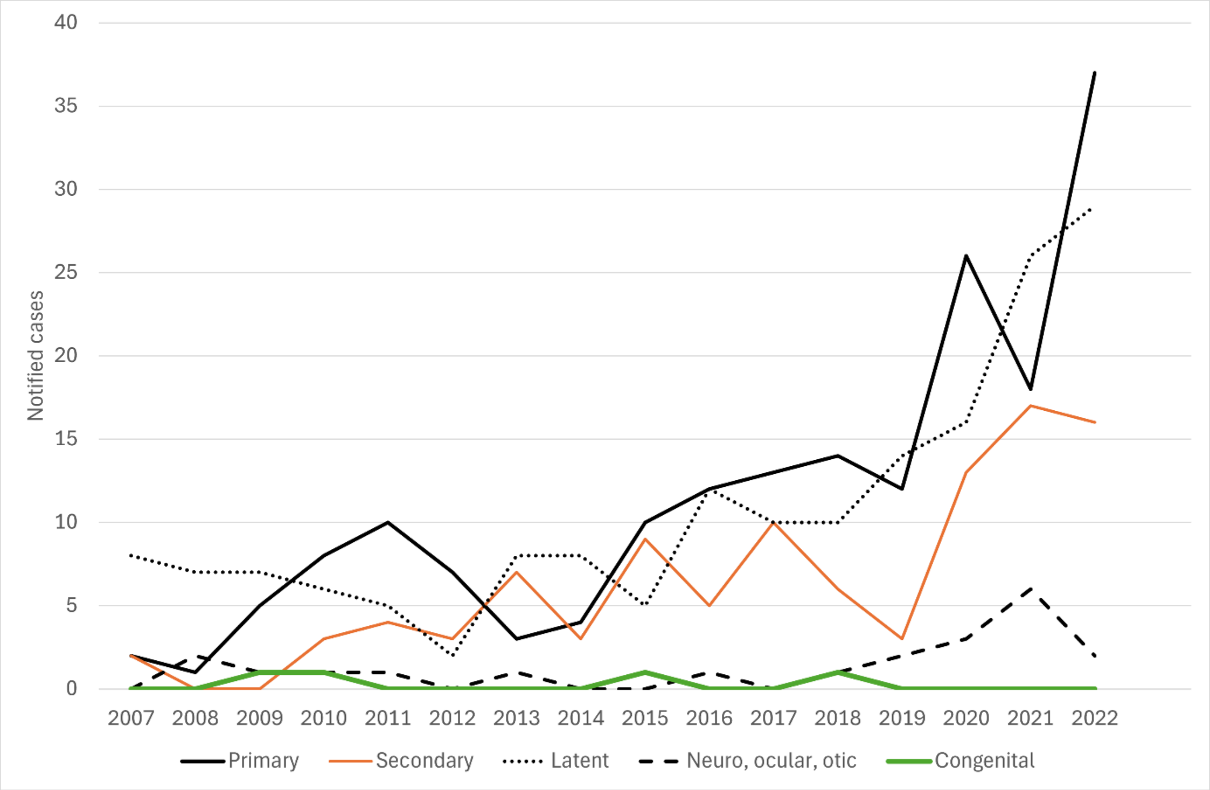

Supplemental figure 2. Number of notified syphilis cases per reported syphilis stage in Skåne region 2007-2022.

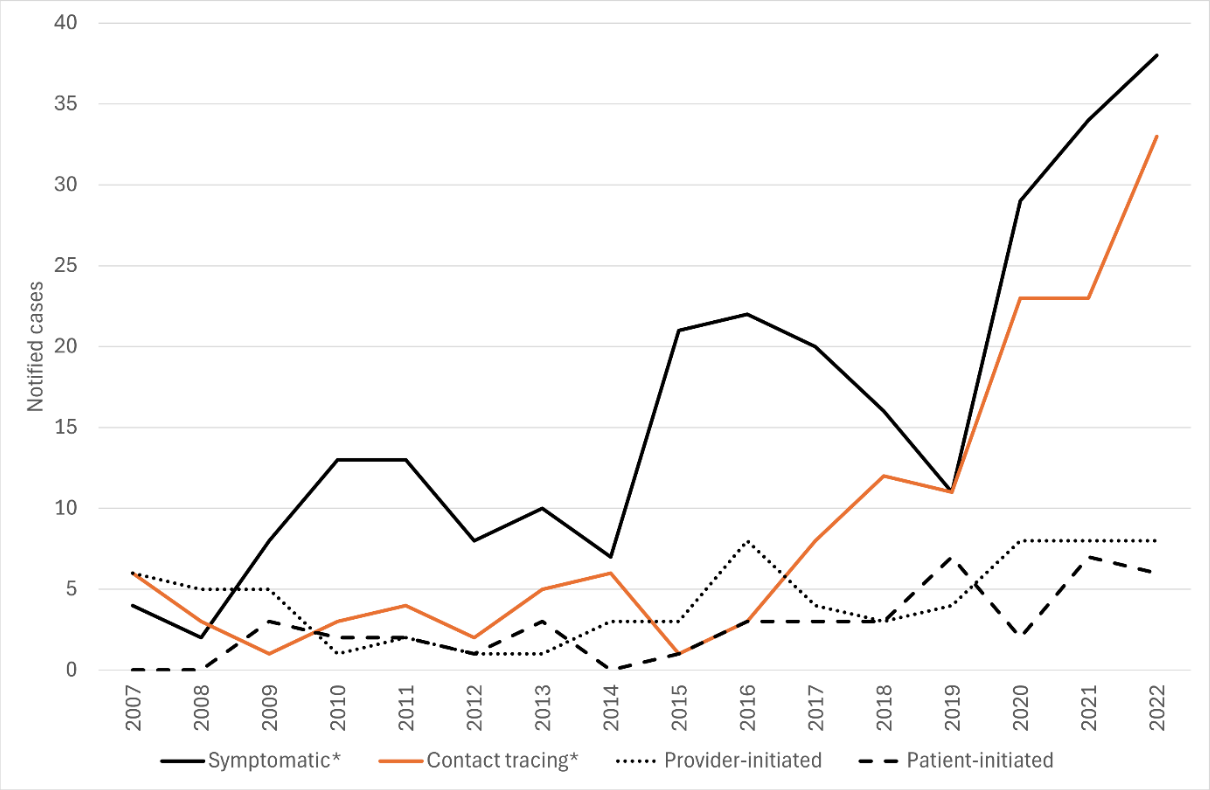

Supplemental figure 3. Number of notified syphilis cases per reported indication for test in Skåne region 2007-2022. *) Statistically significant increasing trend, p<0.01

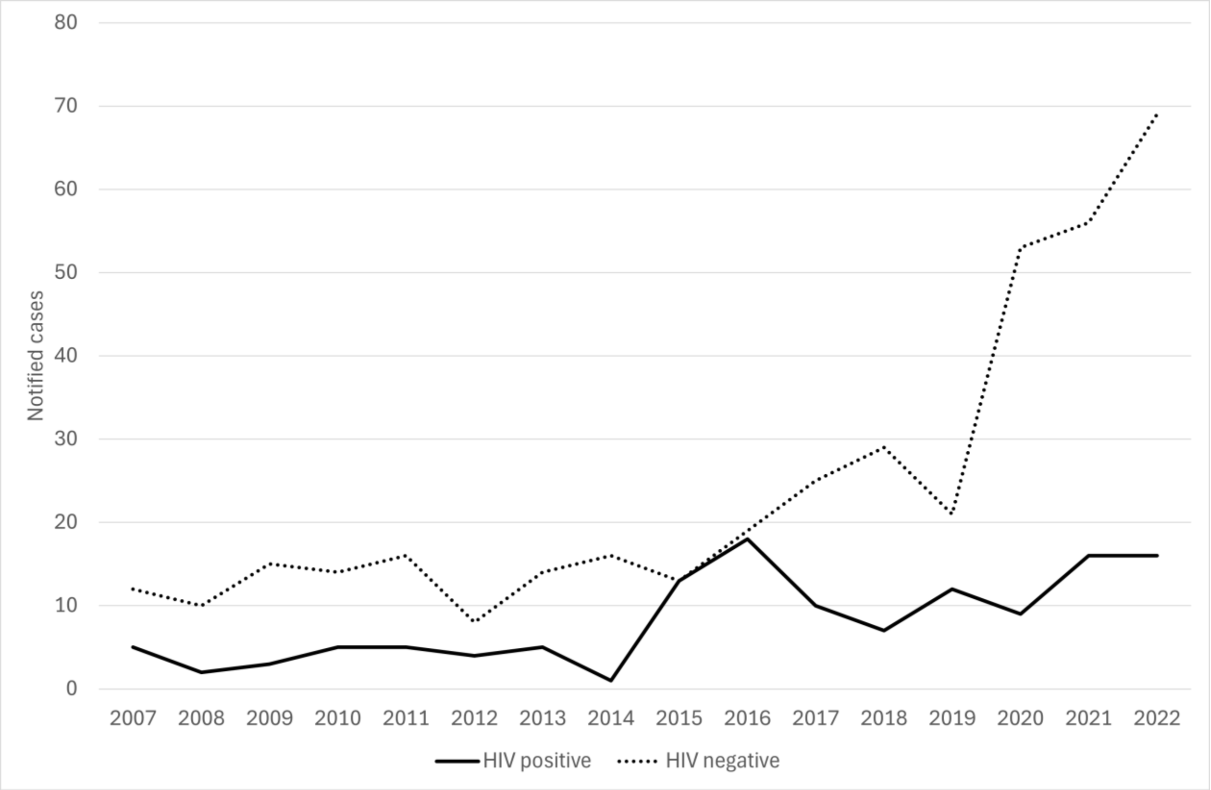

Supplemental figure 4. Number of notified syphilis cases per HIV status at time of diagnosis 2007-2022.
